# Measuring Speech Intelligibility with Romanian Synthetic Unpredictable Sentences in Normal Hearing

**DOI:** 10.3390/audiolres14060085

**Published:** 2024-12-01

**Authors:** Oana Astefanei, Sebastian Cozma, Cristian Martu, Roxana Serban, Corina Butnaru, Petronela Moraru, Gabriela Musat, Luminita Radulescu

**Affiliations:** 1 Doctoral School, Grigore T Popa University of Medicine and Pharmacy, 700115 Iasi, Romania; sebastian.cozma@umfiasi.ro (S.C.); moraru.petronela-ana-maria@d.umfiasi.ro (P.M.); luminita.radulescu@umfiasi.ro (L.R.); 2 ENT Clinic Department, Clinical Rehabilitation Hospital, 700661 Iasi, Romania; martu.cristian@umfiasi.ro (C.M.); roxana-n-serban@umfiasi.ro (R.S.); butnaru.corina@umfiasi.ro (C.B.); 3 Department of Otorhinolaryngology, Faculty of Medicine, Grigore T Popa University of Medicine and Pharmacy, 700115 Iasi, Romania; 4 Department of Otorhinolaryngology, “Carol Davila” University of Medicine and Pharmacy, 050474 Bucharest, Romania; gabimusat@yahoo.com

**Keywords:** Romanian speech, sentence in noise, speech recognition threshold, text-to-speech system, synthetic speech

## Abstract

Background/Objectives: Understanding speech in background noise is a challenging task for listeners with normal hearing and even more so for individuals with hearing impairments. The primary objective of this study was to develop Romanian speech material in noise to assess speech perception in diverse auditory populations, including individuals with normal hearing and those with various types of hearing loss. The goal was to create a versatile tool that can be used in different configurations and expanded for future studies examining auditory performance across various populations and rehabilitation methods. Methods: This study outlines the development of Romanian speech material for speech-in-noise testing, initially presented to normal-hearing listeners to establish baseline data. The material consisted of unpredictable sentences, each with a fixed syntactic structure, generated using speech synthesis from all Romanian phonemes. A total of 50 words were selected and organized into 15 lists, each containing 10 sentences, with five words per sentence. Two evaluation methods were applied in two sessions to 20 normal-hearing volunteers. The first method was an adaptive speech-in-noise recognition test designed to assess the speech recognition threshold (SRT) by adjusting the signal-to-noise ratio (SNR) based on individual performance. The intelligibility of the lists was further assessed at the sentence level to evaluate the training effect. The second method was used to obtain normative data for the SRT, defined as the SNR at which a subject correctly recognizes 50% of the speech material, as well as for the slope, which refers to the steepness of the psychometric function derived from threshold recognition scores measured at three fixed SNRs (−10 dB, −7 dB, and −4 dB) during the measurement phase. Results: The adaptive method showed that the training effect was established after two lists and remained consistent across both sessions. During the measurement phase, the fixed SNR method yielded a mean SRT50 of −7.38 dB with a slope of 11.39%. These results provide reliable and comparable data, supporting the validity of the material for both general population testing and future clinical applications. Conclusions: This study demonstrates that the newly developed Romanian speech material is effective for evaluating speech recognition abilities in noise. The training phase successfully mitigated initial unfamiliarity with the material, ensuring that the results reflect realistic auditory performance. The obtained SRT and slope values provide valuable normative data for future auditory assessments. Due to its flexible design, the material can be further developed and extended to accommodate various auditory rehabilitation methods and diverse populations in future studies.

## 1. Introduction

Speech audiometry involves listening to words or sentences in quiet or in the presence of a competing noise. The results obtained with sentences are more representative of the listener’s performance in real-life situations [1,2]. The speech presented in noise measures has greater utility, as it relates more closely to the measurement of perceived auditory disability [1,2,3]. Speech-in-noise tests are subjective psychoacoustic measurements of speech intelligibility when a competing noise is delivered simultaneously. The intelligibility of speech can be evaluated according to the signal-to-noise ratio (SNR), i.e., the difference in intensity between speech presentations and noise, rather than by the absolute value of recognition thresholds, as it is a better measure of the functionality and performance of the auditory system [3,4]. The intelligibility can be displayed by SNR as a logistic function with a maximum intelligibility of 100% in normal hearing subjects. This function can be characterized by speech recognition threshold 50 (SRT50) and the slope. SRT50 is the level difference between the speech signal and competing noise required for a person to understand 50% of the speech material and slope is the steepness of the curve, indicating how quickly recognition improves with increasing SNR. An x% slope means that for each 1 dB increase in SNR, the percentage of correctly recognized words increases by x%, representing the gain per unit SNR [3,4].

The necessity of understanding whether a certain patient could benefit from the current hearing technology, as well as quantitatively determining the functional improvement levels, has promoted the integration of better hearing diagnostic methods [5]. Since the outcomes of speech audiometry are used to decide on amplification with hearing aids, cochlear implantation, or rehabilitation strategies, it is very crucial that the speech intelligibility assessment is performed with valid and reliable methods [6]. The validation of speech tests requires several steps, including the development and optimization phase (the acoustic energy must be equalized between the speech stimuli) and the evaluation and validation phase (gathering normative data for different test procedures, different listening conditions, and/or types of different hearing loss conditions for the training effect and the test-retest variability) [4,7,8].

In order to highlight some changes in the ability to understand speech-in-noise, a sufficient number of sentence lists is required. Natural, meaningful sentences are subject to redundant effects that can only be removed by using a large enough number in order not to be recognized from the context or based on a previous presentation. These are closer to natural speech in a given language but are more difficult to produce. Unpredictable sentences based on a fixed structure can be produced in sufficiently large numbers, they are perceptually equivalent, but it is necessary to consider the training effect and the redundancy (the same words several times within a trial).

The matrix sentence test is a speech-in-noise assessment designed to determine the speech reception threshold (SRT) with a precision of ±1 dB [6,7,8]. The sentences in the matrix test share the same syntactic structure and are grammatically correct but are semantically unpredictable. This test is based on Hagerman sentences, which consist of 50 words selected from a core vocabulary, enabling the creation of 100,000 unique sentence combinations. Using this approach, matrix tests have been developed for 19 languages, employing similar methodologies across them. In 2015, the International Collegium of Rehabilitative Audiology (ICRA) published guidelines for producing sentence or digit matrix tests in various languages. These guidelines recommend that evaluation and validation should be conducted using either a fixed or adaptive procedure based on 50% or 80% word recognition thresholds. To ensure equivalence across test lists, at least two signal-to-noise ratios (SNRs) corresponding to 20% and 80% intelligibility should be measured for each test list. Additionally, it is recommended to report the standard errors associated with the SRT and slope estimates [7,8,9,10].

Functional hearing refers to an individual’s ability to utilize auditory information to process speech in noisy or otherwise challenging listening environments. The speech-in-noise (SIN) test, available through AC440 module from Affinity2.0/Equinox2.0, (version 2.12.3), Interacoustics (Middelfart, Denmark) (which can be used with any speech material input into the software), “is a method for assessing functional hearing in the presence of competing sounds. The goal of the SIN test is to determine the functional hearing capacity of the subject: specifically, their ability to distinguish between relevant (speech/signal) and irrelevant (noise/masking) auditory information. The test provides the speech recognition threshold (SRT) across a range of signal-to-noise ratios (SNRs). The SIN test is designed for administration via loudspeakers in a free-field setupʺ [11]. This test adheres to ICRA’s recommendations for adaptive SRT evaluation by averaging the SNRs from consecutive trials, though it employs a 3 dB step change in SNR rather than the recommended 2 dB steps [7].

Given the substantial time and resources required for the development of speech tests in noise, Neusse (2019) and Ibelings (2022) investigated the validity of using synthesized speech to generate speech material [12,13]. They compared speech intelligibility thresholds for natural female speech (Oldenburg and Göttingen tests) with those obtained from the same phonetic material produced by a female voice from a text-to-speech (TTS) system. The results revealed minimal differences between the two speech variants. Specifically, the speech recognition thresholds were 0.5 dB higher (worse) for synthetic speech, while the slopes of the functions were comparable for both speech types [12]. The validity of using synthetic speech has thus been established, in line with the impressive advancements in neural network-based speech production systems. Moreover, the optimization stage—formerly necessary for measuring and adjusting the intelligibility of individual words—is no longer required thanks to the sophisticated processes involved in the creation of synthesized voices. The speech material is subjected to complex lexical and acoustic analyses, with the optimization and equalization of vocal production already incorporated into the synthesis process [12,13].

Romanian is a Romance language that evolved from various dialects of Vulgar Latin between the 5th and 6th centuries. It is the official language of Romania and the Republic of Moldova, and it is widely spoken by the Romanian diaspora. Approximately 25 million people speak Romanian worldwide. While Romanian shares many linguistic features with other Romance languages, it also exhibits distinct characteristics influenced by Slavic, Turkish, Hungarian, French, and German. Its lexical similarity is closest to Italian, followed by French, Sardinian, Catalan, Portuguese, and Spanish. Contemporary Romanian does not have distinct dialects.

For Romanian language, there is a clinically validated material consisting of single words, currently used in the clinic battery [14], but we are not aware of the existence of any speech test based on sentences presented in noise. The primary objective of this study was to develop speech material presented in noise (sentence lists) for the Romanian language, which is essential for evaluating speech perception performance and assessing outcomes in patients undergoing various types of auditory rehabilitation [1,2,4,15,16,17].

## 2. Materials and Methods

The speech material was selected to ensure the representation of Romanian phonemes in unpredictable sentences with a fixed syntactic structure [8]. A text-to-speech (TTS) technology was then employed to generate the speech material using a conversational female voice. The evaluation was conducted in the training phase using an adaptive signal-to-noise ratio (SNR) method, employing the speech-in-noise (SIN) test for functional speech reception threshold (SRT) at the sentence level. The objective was to quantify the training effect specific to the speech material and to assess the psychoacoustic equivalence (intelligibility) between test lists. Subsequently, in the measurement phase, SRT50 and slope values were determined using a fixed-level SNR method (three SNR levels) to obtain normative data at the word-scoring level.

### 2.1. Selecting Words for Each Category of Phonetic Material

For the construction of a phonemically balanced list, “a list containing approximately in equal proportions the variety of phonemes present in a spoken communication” [18], information regarding Romanian phonemes was searched in the available literature. Some authors describe a modern phonological system of the Romanian language of 7 vowels, 4 semivowels, and 20 consonants [19,20]. Other authors include another 2 consonants (the velars /c/ and /ɟ/ are taken as separate phonemes) [21,22]. Moreover, TTS systems take into account all allophone variants using a number of 31 phonemes in the production of synthetic speech. From an acoustic point of view, the peculiarities of the Romanian phonemes like specific vowels, (such as ă = /ɔ/, î, and â = /ɪ̃/), give the Romanian language a distinct acoustic profile, while the presence of fricative /ts/ and /dʒ/ before /e/ or /i/ are remarkable for their clarity in pronunciation. The vowel /i/ has a special status depending on the position in the word. In the word /ˈver.zʲ/, the sound /ʲ/ is a palatalization of the previous consonant. Phonetically, it does not have a separate articulation, being considered a consonant /i/. We also included the production /j/ from /ʒuˈkərij/ and /joˈnut͡s/ for semivowel status. We signal some constructions specific to the Romanian language. For example, /ʧinʧʲ/ has 4 phonemes: in the first position, /i/ has syllabic value; in the second position, it has only the value of a diacritical mark.

In order to create the speech material, a fixed sentence structure consisting of five positions (name, verb, numeral, noun [object], adjective) was established, following the model of Hagerman sentences or matrix tests. This structure reflects the syntactic order of words in affirmative sentences in the Romanian language. For each position, 30 frequently used words were selected from the core vocabulary, as listed in the Dictionary of the Romanian Academy. The aim was to create a selection base for each grammatical category. The frequency of occurrence (greater than 50 occurrences) was verified using the COROLA language corpus, which serves as the reference corpus for contemporary Romanian. The COROLA corpus was developed by the Romanian Academy in collaboration with the Artificial Intelligence Research Institute in Bucharest and the Theoretical Informatics Institute in Iași [23]. This corpus is publicly available for text and audio data search via two interfaces. Selection of words adhered to phonological and grammatical criteria to ensure proper agreement between the different parts of speech. Any possible combination of words from these categories results in grammatically correct but semantically neutral and unpredictable sentences (Table 1).

This methodology ensures that the speech material is well-structured, linguistically valid, and suitable for testing speech recognition in noisy conditions. Accordingly, 10 words were selected for each grammatical category. Common masculine proper names were chosen, specifically those ending in consonants (excluding names ending in -a, which are typically feminine). For the numeral category, gender was assigned based on the associated noun (e.g., the numeral două [two] is feminine, so it was paired with feminine or neuter nouns and adjectives). The adjective category included words denoting colors or qualities. Each word category was limited to 2 or 3 syllables, with only three exceptions where a word had a single syllable.

### 2.2. Text-To-Speech System (TTS), Creation of Files, Audiometric Testing System

A web search was conducted for TTS systems that provide female voices in Romanian. Five systems that have the ability to adjust the speech rate and fundamental frequency in the demo were selected. Nine sentences from a third-grade “Natural Sciences book” were entered into each TTS system, and the resulting files were downloaded with the system and voice name. The standard settings for all systems were used, and the presentation of the material was done via a personal computer. Five native Romanian speakers listened and rated each file twice (in a quiet room and in a hospital ward). The mean opinion score (MOS) rating was used, where 5 = perfect, 4 = good, 3 = average 2 = unsatisfactory, 1 = very bad. MOS evaluation reflects the perceived quality of sound by end users in the telecommunications industry. The choice was based on the score of each free available system: Lisntr AI (4.06), Play ht 4 (4.02), Narakeet Alina (4.9), Narakeet Carmen (4.36), and Google Voice (4.36). The features assessed were the natural intonation, intelligibility, correct stress, and co-articulations between phonemes and between words in the sentences.

Based on the selected 50 words, 150 sentences were created and entered into Narakket’s TTS system for Alina’s voice. The standard settings were used for speech rate and pitch, and each sentence was generated as a single WAV file (mono audio files with a sampling rate of 48 kHz, 38 bits). These files were doubled to stereo with a sampling rate of 16 bits, 44.1 kHz by processing the material in Audacity (version 3.0.2), a freely available software. A digital normalization was performed for root mean square (RMS) value of audio signal to −25 dBFS (total digital decibels) of each separate sentence at the suggestion of the developers of the synthetic voice test. Normalizing the RMS ensures that all sound samples have a standardized and comparable intensity level. In the end, the total material has the same RMS level. The speaking rate was between 3.38–4.28 syllables/s. In order to play the sentences through a clinical audiometer, a speech extraction tool, Interacoustics, was used. All material was entered into the Equinox 2.0 audiometer software, version 2.12.3, by individually selecting sentences into separate lists. A 500 ms nsegment of silence was inserted at the beginning and end of each sentence during processing by the extraction tool. Finally, each list had 10 sentences containing all of the selected 50 words. No sentence was repeated in any list, and no word was repeated within a listvThe background noise used was the stationary speech noise provided by the audiometer manufacturer [11]. The Speech Noise used in audiometric testing is specifically designed to replicate the frequency characteristics of normal speech.

### 2.3. Subjects, Test Set-Up, and Parameters

The measurements were conducted in a sound-attenuating semi-anechoic chamber (with walls and floor lined with absorbent materials), specifically prepared for free-field testing. The room had an average background noise level of 41.05 dBA, measured with a PCE-322A sound pressure level meter (measurement range: 30 dB to 130 dB, resolution: 0.1 dB). The subjects were 20 volunteers aged between 19 and 30 years (mean age: 24.95 ± 3.14 years), consisting of 12 women and 8 men. They are originally from different areas (urban and rural) of North-Eastern Romania, and half of them have higher education. All subjects had hearing thresholds of less than 10 dB for all frequencies between 125 Hz and 8000 Hz in both ears. The equipment calibration was performed by an authorized partner, and the calibration certificate (No. 3816) was issued on 11 December 2023. The stimuli were presented through the audiometer using 44,100 Hz, 16-bit, 2-channel sampled audio files. The noise was presented at a constant sound pressure level (SPL) of 65 dB. Two experimental sessions were conducted, each consisting of a training phase and a measurement phase. During the training phase, stimuli were presented through a single calibrated speaker placed 1 m in front of the subject at head level. In the measurement phase, stimuli were presented through equivalent free-field headphones (TDH39) to the right ear. 

The technical specifications included in the Affinity 2.0/Equinox 2.0 manual [11] comply with the established standards set forth by professional associations such as the International Electrotechnical Commission (IEC) and the American National Standards Institute (ANSI).

In both sessions, a random list was initially presented in silence to ensure recognition of the entire speech material. For the training phase, list No. 15 was presented first, followed by lists No. 1 to No. 7 with the speech-in-noise (SIN) test. The software played the first sentence at a signal-to-noise ratio (SNR) of 0 dB (fixed noise level of 65 dB SPL) and then the SNR was adjusted by ±3 dB depending on whether all five words of the sentence were correctly recognized (sentence scoring).

In the measurement phase of the first session, an additional 8 lists (from list No. 8 to No. 15) were presented at SNRs of −4 dB, −10 dB, and −7 dB (with a constant noise level of 65 dB SPL). Responses were manually recorded by a research team member for each word of each list at each SNR, with a score of 1 (for recognition) or 0 (for non-recognition).

**Figure 1:**
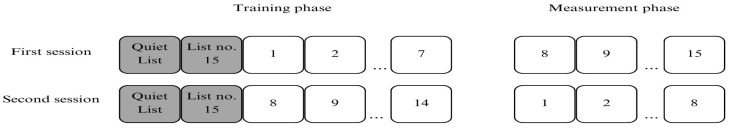
Diagram of the experiment divided into a training phase and a measurement phase. Numbers 1 to 15 represent the number of lists in random order, not the list titles, except for List 15 in the training phase. In Session 1, the training phase includes Lists 1 to 7, and the measurement phase includes Lists 8 to 15. In Session 2, the training phase includes Lists 8 to 14, and the Measurement Phase includes Lists 1 to 8. These lists were presented in random order.

The second session was scheduled between 1 and 10 days after the first session (median 7). It began with a random list in silence, followed by list No. 15 in noise, and continued with the training phase (lists No. 8 to No. 14). The measurement phase in the second session involved presenting lists from No. 1 to No. 8. Each session lasted between 60 and 90 min. The lists were presented in random order within each session and between listeners (Figure 1). The sentences within the list were presented in the same order.

To obtain word-specific intelligibility functions, speech intelligibility was measured at fixed SNR chosen to cover a broad range of intelligibility from approximately 20% to 80% in the measurement phase [4,6,9,10]. The word scores obtained were used to determine speech intelligibility functions by listeners, word lists, and word categories. The average speech intelligibility probability for words presented in a sentence test was estimated by fitting a logistic function (Equation (1)):

(1) $${SI}_{word}\left(SNR\right)=\frac{100}{1+e^{4{S50}_{word}({SRT}_{word}-SNR)}}$$

where SI_word_ = the speech recognition in percent, SRT_word_ = word-specific SRT, and S50_word_ = slope at the SRT_word_ [4,6,18,19].

In this study, we utilized the following parameters with their respective notations:

SNR (Signal-to-Noise Ratio): The ratio between the speech signal and background noise, measured in decibels. In this study, the signal refers to the speech material.

SRT50 (Speech Recognition Threshold 50%): The signal-to-noise ratio at which the subject correctly recognizes 50% of the speech material, expressed in decibels SNR. This refers to the threshold of speech recognition in the presence of background noise, not an absolute speech threshold.

Slope: The slope of the psychometric function derived from the measurement of speech recognition scores across different SNR levels. The slope indicates how steeply the recognition scores change as the SNR is adjusted.

SRT Functional: The speech recognition threshold at which the subject recognizes 50% of the speech material, expressed in decibels SNR, as determined by the speech-in-noise (SIN) test by Interacoustics.

### 2.4. Statistical Analysis

The descriptive statistics were calculated (the mean value ± standard deviation) after checking the normality of the data. The differences between speech recognition score, lists, listeners, words, and value for SRT50 were tested with the ANOVA. Estimations were performed using Stata MP 16 and Python 3.13 software packages. The *p*-value < 0.05 was considered significant for all comparisons. The effect size was calculated as eta squared and Cohen model [24,25].

## 3. Results

### 3.1. Phonetic Material

**Figure 2:**
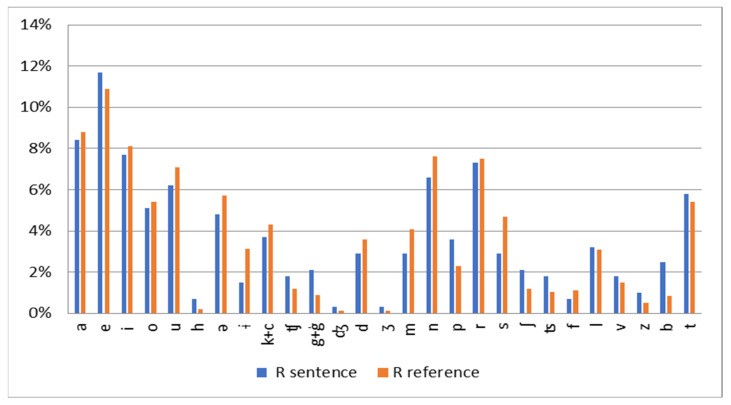
Phoneme frequency distribution in the Romanian speech material (represented in blue) compared to the reference phoneme frequency distribution for the Romanian language (represented in red). The phonemes are transcribed using International Phonetic Alphabet (IPA) symbols.

The Romanian language consists of 7 vowel sounds and 22 consonant sounds, corresponding to its phonemes. In the selected material, which contains 274 phonemes, 150 were consonants (54.74%) and 124 were vowels (45.26%). These percentages align with the reference frequencies for consonants (ranging from 49.74% to 54.84%) and vowels (ranging from 45.16% to 49.40%) reported in the literature. To compare the selected material with established phoneme frequency data, two pairs of phonemes—/ģ/ and /g/, as well as /k’/ and /k/—were grouped together due to their low frequency (less than 1%). Additionally, semivowels, which occur in diphthongs, were treated as vowels for the purposes of this analysis. The phoneme frequencies for the selected material were transcribed using the International Phonetic Alphabet (IPA) and compared with reference data from previous studies to assess their consistency with known patterns of phoneme distribution in Romanian (Figure 2).

### 3.2. Speech Recognition at Different SNRs, the Psychometric Function with Fixed SNR

The data from both sessions during the measurement phase were used to calculate the speech recognition scores for a total of 900 observations (15 lists × 20 listeners × 3 SNR levels). A normal distribution was confirmed for the speech recognition scores based on the Jarque–Bera test for normality (H_0_ = normality, *p* > 0.5).

A one-way ANOVA was conducted to assess the effect of SNR level on speech recognition scores. The results confirmed a significant difference in speech recognition across the three SNR levels, F(2, 897) = 30,683.7, *p* < 0.001. Further, one-way ANOVA was performed separately for each SNR level between lists. At SNR −10 dB, significant statistical differences were observed between lists (F = 2.07, *p* = 0.0137). However, no significant differences were found for SNR −4 dB (F = 1.47, *p* = 0.1219) and SNR −7 dB (F = 0.69, *p* = 0.7843). The highest standard deviation in intelligibility scores was observed at SNR −10 dB, with the largest differences in performance between List 12 and List 2 (Table 2).

**Figure 3:**
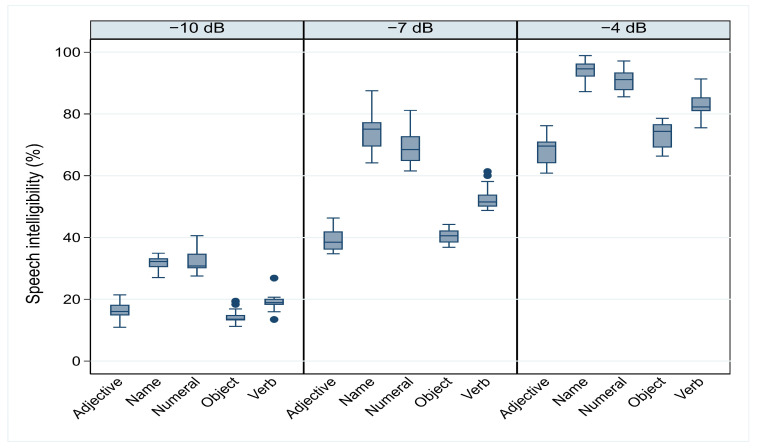
Speech recognition scores for all listeners across different word categories at the three signal-to-noise ratios (SNRs) from all measurement lists. The horizontal lines inside the boxes represent the median values, while the lines extending from the boxes indicate the minimum (lower) and maximum (upper) values. The circles represent outliers. The interquartile range (IQR) is represented by the width of the box, which spans from the first quartile (Q1) to the third quartile (Q3).

Figure 3 shows the box plots for the speech recognition scores across three SNR levels (SNR −10, SNR −7, and SNR −4) and for each word category (verbs, adjectives, names, numerals, and objects). The box plots provide a visual comparison of the distribution of values across conditions and categories, allowing us to assess the variability and central tendency of the scores.

In the SNR −10 condition, characterized by a high level of background noise, scores for verbs and objects show relatively small dispersion, with most values concentrated around the median. Despite the presence of two outliers in each category, the data suggest consistent recognition performance, although with lower overall scores. In contrast, adjectives and names show a more symmetric distribution, with medians located near the center of the boxes. However, names show a slight skew toward higher values, with Q1 very close to the median and the maximum further away from Q3, suggesting a slightly better performance for names compared to other categories.

For the SNR −7 condition, names and numerals still yield the best recognition scores, similar to the SNR −10 condition, but with greater dispersion. Two outliers were observed for verbs, indicating more variability in recognition scores for this category. In addition, for names, numerals, and adjectives, the upper quartile (Q3) is slightly longer, suggesting a general trend toward higher recognition scores. On the other hand, objects show smaller dispersion, and the distribution remains nearly symmetric, similar to the SNR −10 condition.

In the SNR −4 condition, which represents the easiest recognition condition, a symmetric distribution is observed for most word categories. The whiskers are relatively equal for all categories, and the medians are positioned towards the upper part of the boxes, indicating higher recognition scores compared to the noisier conditions. Specifically, names and numerals have nearly symmetric distributions, with names showing slightly better performance. Verbs and adjectives also show higher scores than in the previous condition but remain lower compared to names and numerals. For objects, the distribution is nearly symmetric with a slightly longer box, indicating relatively better performance in this condition compared to the more challenging SNR conditions.

Statistically significant differences were found between word categories, as revealed by a repeated-measures two-way ANOVA. The assumption of sphericity was violated, as indicated by significant values for Mauchly’s test of sphericity (χ^2^ = 53.21, *p* < 0.001 for SNR −4; χ^2^ = 49.11, *p* < 0.001 for SNR −7; and χ^2^ = 24.22, *p* = 0.00412 for SNR −10). Greenhouse–Geisser corrections were applied, and post hoc comparisons revealed significant differences between word categories, except for a few cases where no significant differences were found (e.g., between objects and adjectives at SNR −7 and −10 and between verbs and adjectives at SNR −10) (see Table 3).

**Figure 4:**
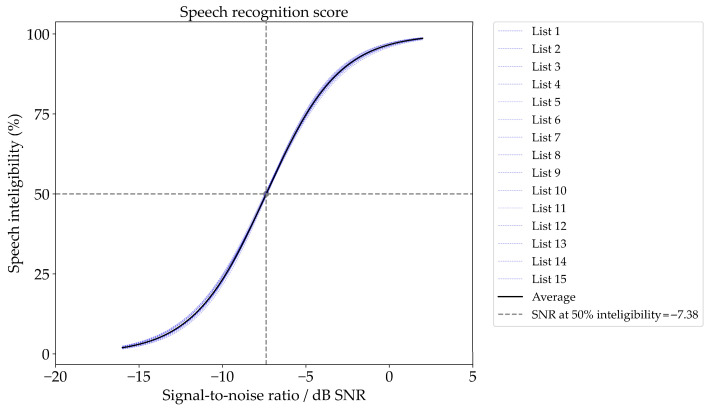
Individual psychometric functions for each list are in dashed blue lines. The mean psychometric functions with average curve parameters are shown as black line.

The logistic regression model applied to the data across all lists and listeners yielded a mean speech reception threshold (SRT50) of −7.38 dB and a slope of 11.39%. The slope across the lists was estimated at 11.40%/dB ± 0.39, and the threshold was −7.38 dB ± 0.05 (Figure 4). When parameters were estimated across the listeners, the mean SRT was −7.38 dB ± 0.12, and the slope was 11.28% ± 0.46. These estimates provide a consistent overview of speech intelligibility performance under varying noise conditions.

**Figure 5:**
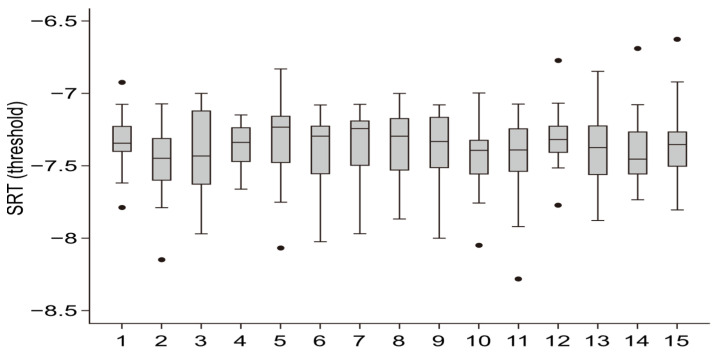
SRT50 in dB SNR across 20 listeners for 15 measurement lists. The lines inside the boxes represent the mean, while the lines extending from the boxes indicate the minimum and maximum values. Circles represent outliers, and the interquartile range (IQR) is shown by the width of the box, spanning from the first quartile (Q1) to the third quartile (Q3).

The analysis of the SRT50 values across the 15 measurement lists reveals moderate dispersion, with interquartile ranges (IQRs) ranging from 7.1 to 7.6 dB (Figure 5). Outliers were observed in some of the lists, although these outliers did not exert a significant influence on the central tendency or overall distribution of the data. A box plot analysis confirmed that the SRT50 values were consistently distributed across the measurement lists, suggesting that the test material was equivalent in difficulty across lists. This was further supported by the results of a one-way ANOVA, which showed no statistically significant effect of the measurement lists on the SRT50 values (F(14, 885) = 0.02, *p* = 1.000). These findings suggest that the variation in SRT50 scores observed across the lists is minimal and does not affect the overall reliability of the test.

### 3.3. Measuring Speech Intelligibility with Adaptive SNR Procedure

**Figure 6:**
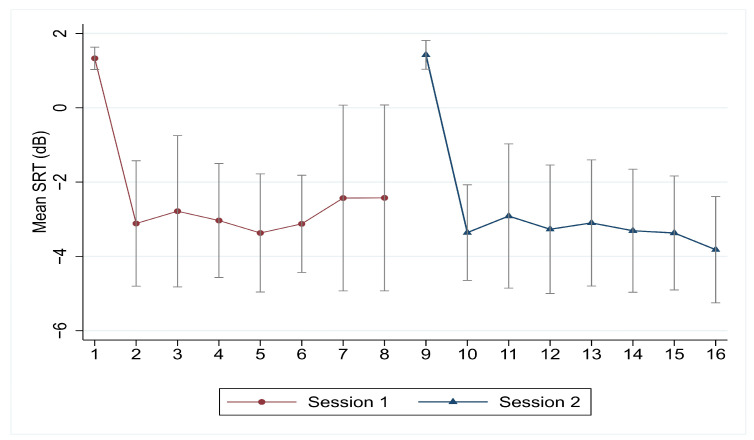
The mean SRTs for training phase (eight lists in each session) using the adaptive procedure. Training was randomized between test lists. The number of the lists represents the order of presentation and not the title of the lists with the exception of List 15, which was presented first in noise in both training sessions.

The significant statistical difference between the training lists is confirmed when running repeated measures of two-way ANOVA to explore differences between the lists across the listeners for each of the two sessions. Mauchly’s test indicated that the assumption of sphericity is rejected for each of the two sessions when the complete set of training lists was included (eight lists for each session (for Session 1: χ^2^(27) = 56.35, *p* = 0.001, for Session 2: χ^2^(27) = 55.36, *p* = 0. 0.00129)). Given that sphericity is rejected, the Greenhouse–Geisser corrected tests are reported (epsilon = 0.647 for Session 1 and 0.634 for Session 2), which indicate significant differences between the SRT across the lists for both sessions, with F(7, 133) = 13.16, *p* < 0.001 for Session 1 and F(7, 133) = 22.24, *p* < 0.001 for Session 2. However, when graphically investigating the average functional SRT across the lists, Figure 6 clearly points out that the largest change in SRT occurs between the first and the second list in each of the two sessions (the drop is about 4.45 and 4.49 dB for Session 1 and Session 2) (Table 4).

Thus, if we remove List 15 (first presented in noise) from each of the sessions, the results no longer indicate a significant statistical difference between the remaining (seven) lists. The sphericity hypothesis is also no longer rejected and the unadjusted statistics of repeated measures of a two-way ANOVA are F(6, 114) = 0.65, *p* = 0.6934, and F(6, 114) = 0.52, *p* = 0.7884.

The statistical differences between the SRT across the lists for each session were also confirmed when multiple post hoc tests were computed (accounting for the Bonferroni correction). In the same vein as the results reported above, only the SRT in the first list differs from the other lists in each of the two sessions, but without significant differences between the rest of the lists (see Table 5 and Table 6).

The effect size, calculated as eta squared, was 0.398 for Session 1 and 0.532 for Session 2, indicating a large effect of lists on SRT for both sessions. Similarly, the Cohen’s f effect size is 0.813 (Session 1) and 1.066 (Session 2), which confirms the strong effect. When we drop the first list from each of the sessions, the effect becomes smaller in size (eta squared is 0.031 for Session 1 and 0.026 for Session 2). The Cohen’s f effect size also decreases to 0.179 (Session 1) and 0.163 (Session 2).

## 4. Discussion

The need for this study stems from the importance of accurate evaluation of speech recognition in noise. This study aims to evaluate a newly developed Romanian speech material and ensure its reliability across different testing conditions. Specifically, we focused on speech recognition performance across two phases: the training phase (to evaluate the learning effect) and the measurement phase (to assess SRT50 and slope at fixed SNRs). By comparing the performance with the existing literature, we can better understand the efficacy and potential of the developed material. The 50 words were selected from a pool of frequently used words for five grammatical categories, based on the core vocabulary. Words were chosen based on their frequency and adherence to phonological and grammatical criteria, ensuring proper agreement between different parts of speech.

### 4.1. Methodological Differences Between Studies Using Fixed and Adaptive SNR

Most of the matrix studies combine both adaptive and fixed SNR methods using the adaptive method during the training phase to account for individual performance and allow for learning effects, followed by the fixed SNR method in the measurement phase to ensure precision and consistency in estimating thresholds. In our study, we used only the fixed SNR method to calculate SRT50 and slope, which provided a more controlled approach to measuring speech recognition thresholds. However, the adaptive SNR method (via the SIN test) was employed solely to explore the learning effect during the training phase. This approach allowed us to observe how participants adapted to the test material, but it also meant that we did not capture individual variability across different noise levels in the measurement phase. Although combining both methods in a single design would provide a dynamic measure of performance and more precise thresholds [26], our methodology focused on isolating the learning effect while examining speech recognition at fixed SNR levels.

### 4.2. SRT50 and Slope Analysis

The SRT50 value of −7.38 dB obtained in our study is consistent with results from other studies using the fixed SNR approach. For example, the Italian matrix test reports an SRT of −7.3 ± 0.2 dB (SD across lists) and −7.4 ± 0.9 dB (SD across listeners) [6]. Similarly, the Spanish matrix test reports an SRT of −6.8 ± 0.13 dB [10], and the French matrix test reports an SRT of −6.0 ± 0.6 dB [9]. Our SRT50 value falls within the range of these studies, though it is slightly lower than the French study’s reported threshold. These findings suggest that the speech material and methodology used in our study are comparable to those employed in other languages. However, the slope value of 11.39% in our study is steeper than those reported in the Spanish and French matrix studies, which report slopes of 13.2% and 14.0%, respectively. This steeper slope suggests that listeners in our study exhibited a more sensitive response to increasing background noise levels, possibly due to the more controlled, fixed SNR conditions. The Italian matrix also reports a slope of 13.3%, which is closer to the values seen in the Spanish and French studies, suggesting that variations in listener performance might be minimized when both speech signal and noise levels are dynamically adjusted, as seen in studies using adaptive methods.

The difference in slope values between our study and others could also reflect variability in listener sensitivity. As noted, the fixed SNR method may reduce variability across listeners, as evidenced by the low standard deviation in our estimated slope values (11.28% ± 0.46 across listeners). The Italian test with a roving-level adaptive method (adaptive speech and noise levels) reported a similar SRT50 value of −8.0 dB but with a slightly less steep slope of 11.3% [6]. This supports the idea that adaptive methods can reduce listener variability and yield a more dynamic reflection of performance. Thus, while the results of our study are comparable in terms of SRT, the steeper slope indicates that the fixed SNR method may offer a more refined approach to measuring speech recognition performance in challenging noise environments.

### 4.3. Learning Effect

In our study, the training phase using the SIN test (adaptive SNR method) aimed to explore the learning effect, which was demonstrated by a significant reduction in SRT between the first and second lists presented in noise in both sessions. This rapid improvement is consistent with previous studies, such as the Italian roving-level test matrix, where the learning effect occurred very quickly. However, other studies, including the French, Italian, and Spanish matrix tests, reported that up to three lists were required for the learning effect to be fully established. While some studies have indicated that a larger number of lists were necessary to observe the learning effect, differences in language, as well as the fact that our study used sentence-based scoring rather than word-based scoring, could have contributed to these variations.

### 4.4. Comparing Word Categories and Recognition Performance

Our study also explored the role of word category and position within the sentence on recognition performance, with results showing that adjectives (appearing at the end of sentences) were the most difficult to recognize, especially at lower SNRs (e.g., SNR −10 dB). This finding is consistent with other research, such as that by Neusse et al. [12], who reported that names were the most challenging to recognize in their study. The numerals and names in our study were more easily recognized, particularly in favorable SNR conditions (e.g., SNR −4 dB), supporting the idea that certain categories of words are more salient and familiar in speech processing. These findings align with other studies, such as those by Ahsanul et al. [27], which found that numerals are often the most easily recognized part of speech in noisy conditions. Additionally, in this Romanian corpus, digits showed higher recognition accuracy than other speech categories, especially at lower SNR levels. These observations highlight the importance of word categories in speech recognition, especially in noisy environments. Our results further confirm that the position of words (e.g., adjectives at the end of sentences) plays a significant role in speech intelligibility, particularly under challenging SNR conditions.

Future studies involving clinical populations (e.g., individuals with hearing loss) would help assess how well the test performs in real-world, rehabilitative settings. Furthermore, linguistic and phonetic factors (e.g., word familiarity, phonetic complexity, and sentence structure) should be further explored to refine the test material for better adaptation to various languages and cultural contexts.

### 4.5. Perceptual Equivalence of Lists: SRT50 and Scoring Consistency

An important consideration in speech recognition testing is ensuring that the lists used in the test are perceptually equivalent, meaning that they yield comparable results in terms of both SRT50 and slope. In our study, the SRT50 values calculated for each of the 15 lists were consistent, with standard deviations that remained within a 1 dB margin compared to those reported in matrix studies. This consistency suggests that the difficulty level of the lists was well-matched, and participants’ performance across different lists did not significantly vary in a way that could bias the overall results. The perceptual equivalence of our lists is further validated by the standard deviation of the SRT50 values across the 15 lists, which remained within acceptable ranges when compared to other studies employing the matrix test method.

Furthermore, in terms of scoring at the sentence level, our lists exhibited a similar level of consistency to those based on word recognition. Despite differences in test design, the equivalence of lists at the level of sentence-based scoring was maintained. This suggests that even with the different linguistic structures and sentence complexity used in our study, the test material was equally challenging and reliable for speech recognition assessments.

Overall, the perceptual equivalence between the lists, as measured by both SRT50 and slope values, confirms that the test material provides robust and consistent results. The performance across lists did not vary significantly, supporting the use of this material for further research and clinical applications. The ability to establish equivalence not only in terms of threshold measurements but also in terms of sentence-level scoring reinforces the reliability of the methodology and the validity of the results for assessing speech recognition in noise.

The feasibility of this material for both clinical and research settings is a key aspect of its practical application. The perceptual equivalence of the lists, combined with a sufficient number of lists for training, allows the test to be used effectively in various spatial configurations and under different listening conditions, including aided and unaided conditions, as well as in more complex settings where subjects are required to perform additional tasks. This includes scenarios such as tonal audiometry, localization tests, word lists in quiet, or hearing aid adjustment sessions, where speech-in-noise testing needs to be integrated alongside other assessments.

The combination of adaptive scoring methods and a reduced number of training lists provides a practical and efficient approach for assessing speech intelligibility while maintaining the reliability and perceptual equivalence of the test material. These findings have significant implications for both research and clinical applications, particularly for individuals with hearing impairments, as they suggest that speech-in-noise tests can be optimized for real-world use, reducing the time required for testing while still providing accurate and meaningful results. Future research should continue to explore the impact of word characteristics, test conditions, and scoring methods on speech recognition performance, with an emphasis on applications in environments with varying levels of background noise.

Several limitations of this study should be considered when interpreting the results. Firstly, the relatively small sample size of only 20 volunteers may limit the generalizability of the findings, especially to larger or more diverse populations. Additionally, the study was conducted at a single testing center, which may introduce location-specific biases or limit the external validity of the results. From a methodological standpoint, while we used a fixed SNR approach for measuring SRT50 and slope, combining both fixed and adaptive methods in a single design could have provided a more comprehensive understanding of individual variability in speech recognition performance.

## 5. Conclusions

This study highlights the effectiveness of using a fixed SNR method for measuring SRT50 and the slope of the psychometric function. While our methodology exclusively used the fixed SNR approach for these measurements, the combination of adaptive and fixed SNR methods would likely provide a more comprehensive assessment of speech recognition performance. The adaptive SNR method, used here only to investigate the training effect, adjusts to individual performance, which could yield more dynamic results. Future studies combining both methods would likely achieve more reliable, precise, and individualized performance measures.

The SRT50 value found in our study aligns well with results from other studies using the fixed SNR method, such as the Italian and Spanish matrix tests. Our slope value is somewhat steeper than those reported in other matrix studies (e.g., Spanish, French), suggesting that listeners in our study were more sensitive to the increasing noise levels. This difference in slope values may reflect the methodological differences, particularly the use of the fixed SNR method in our study versus more flexible methodologies (e.g., roving-level SNR) in other studies.

The training phase of our study showed significant improvement in SRT between the first and second test lists in noise, consistent with rapid learning effects observed in other studies. The learning effect can be attributed to participants’ increased familiarity with the speech material during the initial training phase.

Our study found that word category and their position in sentences significantly impacted speech recognition performance. Adjectives, particularly at the end of sentences, were the most challenging to recognize in noisy conditions, especially at lower SNRs. In contrast, numerals and names showed better and more consistent recognition across participants. These findings are consistent with previous research, which highlighted the importance of word salience and position within sentences in determining recognition accuracy.

The perceptual equivalence of the lists in terms of SRT50, slope, and sentence-level scoring demonstrates that our test material is reliable and consistent, ensuring accurate and stable speech recognition assessments across different lists.

Relevance to Clinical Settings: The study’s findings have direct relevance for clinical applications in speech rehabilitation. The ability to reliably measure SRT50 and the slope of the psychometric function provides critical insights into individuals’ speech recognition abilities in noisy environments. These metrics, combined with a better understanding of word categories and the learning effect, can be used to refine speech rehabilitation protocols, particularly for individuals with hearing impairments.

Future Research Directions: To further improve the measurement of speech recognition thresholds, future studies should explore the combination of both adaptive and fixed SNR methods in a single test. Further exploration of word familiarity, phonetic complexity, and sentence structure will enhance the test material’s adaptability across different languages and cultural contexts. This approach could offer a more comprehensive evaluation of speech recognition abilities by accounting for individual variability while still providing reliable thresholds.

The utility of speech material in capturing auditory abilities is paramount for assessing speech recognition across all categories of hearing-impaired subjects, whether rehabilitated or not. Reliable assessment tools are critical not only for determining the baseline abilities of individuals with hearing loss but also for monitoring progress over time. Moreover, speech recognition testing is essential for patients with various types of hearing loss, including bilateral, unilateral, and asymmetric impairments. These differences in hearing loss type can significantly impact speech recognition performance, making it crucial to use adaptable and sensitive measurement techniques in clinical practice.

## Figures and Tables

**Table 1:** The selected words with International Phonetic Alphabet (IFA) transcription between bars. English translation is in the last column. Bolded words represent the formation of a single sentence.

Name	Verb	Numeral	Object	Adjective	English Translation
Mihai/mi’hai/	vinde/‘vin.de/	zero/‘ze.ro/	mașini/‘ma.ʃi.ni/	cuminți/kuˈminʦʲ/	Michael sells zero good cars.
Costel/ˈkos.tel/	anunță/aˈnun.t͡sə/	două/ˈdo.wə/	**scaune/ˈska.u̯ne/**	magice/maˈd͡ʒi.t͡ʃe/	Costel announces two magic chairs
**Florin/floʼrin/**	cumpără/kumpə.rə/	**trei/trej/**	sticle/ˈsti.kle/	**maro/ˈma.ro/**	Florin buys three brown bottles.
Alex/aˈleks/	prinde/ˈprin.de/	patru/ˈpatru/	păpuși/ˈpəpuʃ/	albastre/alˈbas.tre/	Alex catches four blue dolls.
Ștefan/ʃteˈfan/	dorește/doˈreʃ.te/	cinci/ʧinʧʲ/	jucării/ʒuˈkə.rij/	verzi/ˈver.zʲ/	Stephen wants five green toys.
Sandu/ˈsan.du/	**învață/ˈɨn.va.t͡sə/**	șase/ˈʃa.se/	becuri/ˈbe.ku.ri/	galbene/ɡalˈbe.ne/	Sandu learns six yellow bulbs.
Ionuț/joˈnut͡s/	saltă/ˈsal.tə/	șapte/ˈʃap.te/	ghete/ˈɡe.te/	grăbite/ɡrəˈbi.te/	John jumps seven hurry boots.
Bogdan/ˈboɡ.dan/	începe /ˈint͡ʃe.pe/	opt/opt/	haine/ˈhaj.ne/	drăguțe/drəˈɡut͡se/	Bogdan starts eight nice clothes.
Petru/ˈpe.tru/	îmbracă/ˈim.bra.kə/	nouă/ˈno.wə/	vapoare/vaˈpo̯a.re/	violet/vi.oˈlet/	Peter puts on nine purple ships.
Tudor/tuʼdor/	cheamă/ˈkea.mə/	zece/ˈze.t͡ʃe/	globuri/ˈɡlo.bu.ri/	urâte/uˈrɨte/	Tudor calls ten ugly globes.

**Table 2:** SI (speech intelligibility), Slope, and SRT (speech recognition threshold)50 per test list from 20 listeners, by SNR (signal-to-noise ratio) level for the measurement phase.

List	Slope (%)	SRT50	SI (%) by SNR		
			−4 dB	−7 dB	−10 dB
1	11.32	−7.33	81.88	53.73	22.98
2	10.94	−7.47	82.04	55.13	24.84
3	11.81	−7.42	83.42	54.95	22.82
4	11.60	−7.36	82.64	54.21	22.74
5	11.40	−7.33	82.00	53.70	22.80
6	12.02	−7.41	83.72	54.86	22.32
7	11.59	−7.33	82.42	53.86	22.52
8	10.65	−7.36	80.70	53.80	24.50
9	10.98	−7.38	81.55	54.20	24.05
10	11.47	−7.44	82.89	55.02	23.59
11	11.16	−7.43	82.24	54.83	24.14
12	12.03	−7.31	83.07	53.66	21.47
13	10.91	−7.38	81.35	54.10	24.15
14	11.61	−7.39	82.84	54.52	22.94
15	11.52	−7.36	82.44	54.11	22.84
Mean	11.40	−7.38	82.35	54.31	23.25
SD	0.39	0.05	0.77	0.51	0.89

**Table 3:** *p*-values (Bonferroni-corrected for multiple comparisons) of the post hoc pairwise comparisons of SRT by part of speech (within) and listener (between).

	Name	Verb	Numeral	Object
Verb	0.000			
Numeral	0.276	0.000		
Object	0.000	0.000	0.000	
Adjective	0.000	0.000	0.000	0.013

**Table 4:** Summary statistics of functional SRT by list across the listeners in adaptive phase.

Session 1	List 15	List 1	List 2	List 3	List 4	List 5	List 6	List 7
SRT mean	1.33	−3.12	−2.79	−3.04	−3.37	−3.13	−2.43	−2.43
SRT change		4.45	−0.33	0.25	0.34	−0.25	−0.69	−0.01
SRT aggregated change		4.45	4.12	4.37	4.70	4.46	3.76	3.76
Session 2	List 15	List 8	List 9	List 10	List 11	List 12	List 13	List 14
SRT mean	1.43	−3.36	−2.92	−3.27	−3.10	−3.31	−3.37	−3.82
SRT change		4.79	−0.45	0.36	−0.17	0.21	0.06	0.45
SRT aggregated change		4.79	4.34	4.70	4.53	4.74	4.80	5.25

Note: the number of lists represents the order of presentation and not the title of the lists with the exception of List 15 which was presented first in noise in both training sessions.

**Table 5:** *p*-values (Bonferroni-corrected for multiple comparisons) of the post hoc pairwise comparisons of SRTs by list (within) and listener (between), first session.

	List No 15	List 1	List 2	List 3	List 4	List 5	List 6
List 1	<0.001						
List 2	<0.001	1.000					
List 3	<0.001	1.000	1.000				
List 4	<0.001	1.000	1.000	1.000			
List 5	<0.001	1.000	1.000	1.000	1.000		
List 6	<0.001	1.000	1.000	1.000	1.000	1.000	
List 7	<0.001	1.000	1.000	1.000	1.000	1.000	1.000

Note: The number of the lists represents the order in which it was presented and not the number of a test list. The numbering represents the order of presentation of the lists and not the title of the lists with the exception of List 15, which was presented first in noise in both training sessions.

**Table 6:** *p*-values (Bonferroni-corrected for multiple comparisons) of the post hoc pairwise comparisons of SRTs by list (within) and listener (between), second session.

	List No 15	List 8	List 9	List 10	List 11	List 12	List 13
List 8	<0.001						
List 9	<0.001	1.000					
List 10	<0.001	1.000	1.000				
List 11	<0.001	1.000	1.000	1.000			
List 12	<0.001	1.000	1.000	1.000	1.000		
List 13	<0.001	1.000	1.000	1.000	1.000	1.000	
List 14	<0.001	1.000	1.000	1.000	1.000	1.000	1.000

Note: The numbering of the lists represents the order in which it was presented and not the number of a test list. The numbering represents the order of presentation of the lists and not the title of the lists with the exception of List 15, which was presented first in noise in both training sessions.

## Data Availability

The original contributions presented in this study are included in the article. Further inquiries can be directed to the corresponding author.
